# Profiling lysophosphatidic acid levels in plasma from head and neck cancer patients

**DOI:** 10.7717/peerj.9304

**Published:** 2020-06-05

**Authors:** Mariati Abdul Rahman, Didi Erwandi Mohamad Haron, Robert J. Hollows, Zuleen Delina Fasya Abdul Ghani, Mustafa Ali Mohd, Wen Lin Chai, Ching Ching Ng, Munn Sann Lye, Saiful Anuar Karsani, Lee Fah Yap, Ian C. Paterson

**Affiliations:** 1 Department of Oral and Craniofacial Sciences, University of Malaya, Kuala Lumpur, Malaysia; 2 Department of Craniofacial Diagnostics and Biosciences, Universiti Kebangsaan Malaysia, Kuala Lumpur, Malaysia; 3 Department of Pharmacology, University of Malaya, Kuala Lumpur, Malaysia; 4 Institute of Immunology and Immunotherapy, University of Birmingham, Birmingham, United Kingdom; 5 Industrial Biotechnology Research Centre (IBRC), SIRIM Berhad, Selangor, Malaysia; 6 Faculty of Medicine and Health Sciences, UCSI University, Kuala Lumpur, Malaysia; 7 Department of Restorative Dentistry, University of Malaya, Kuala Lumpur, Malaysia; 8 Institute of Biological Sciences, University of Malaya, Kuala Lumpur, Malaysia; 9 Department of Community Medicine, Faculty of Medicine and Health Sciences, Universiti Putra Malaysia, Serdang, Malaysia; 10 Oral Cancer Research and Coordinating Centre, University of Malaya, Kuala Lumpur, Malaysia

**Keywords:** Lysophosphatidic acid, Oral cancer, Nasopharyngeal carcinoma, Lipidomics

## Abstract

Head and neck squamous cell carcinoma (HNSCC) represents a significant world health problem, with approximately 600,000 new cases being diagnosed annually. The prognosis for patients with HNSCC is poor and, therefore, the identification of biomarkers for screening, diagnosis and prognostication would be clinically beneficial. A limited number of studies have used lipidomics to profile lipid species in the plasma of cancer patients. However, the profile and levels of lysophosphatidic acid (LPA) species have not been examined in HNSCC. In this study, a targeted lipidomics approach using liquid chromatography triple quadrupole mass spectrometry (LCMS/MS) was used to analyse the concentration of LPA (16:0 LPA, 18:0 LPA, 18:1 LPA, 18:2 LPA and 20:4 LPA) in the plasma of patients with oral squamous cell carcinoma (OSCC) and nasopharyngeal carcinoma (NPC), together with healthy controls. The levels of three LPA species (18:1 LPA, 18:2 LPA and 20:4 LPA) were significantly lower in the plasma of OSCC patients, whilst the concentrations of all five LPA species tested were significantly lower in plasma from NPC patients. Furthermore, the order of abundance of LPA species in plasma was different between the control and cancer groups, with 16:0 LPA, 18:0 LPA levels being more abundant in OSCC and NPC patients. Medium to strong correlations were observed using all pairs of LPA species and a clear separation of the normal and tumour groups was observed using PCA analysis. In summary, the results of this study showed that the levels of several LPA species in the plasma of patients with OSCC and NPC were lower than those from healthy individuals. Understanding these variations may provide novel insights into the role of LPA in these cancers.

## Introduction

Head and neck cancer refers to a group of tumours that arise in the lip, oral cavity, oropharynx, nasopharynx, paranasal sinuses, pharynx and larynx. Approximately 95% of all head and neck cancers are squamous cell carcinomas (HNSCC), which is the sixth most common cancer worldwide, affecting 600,000 new patients each year with a mortality rate of around 50% (Ferlay et al., 2015; Leemans, Snijders & Brakenhoff, 2018; Rothenberg & Ellisen, 2012). Oral squamous cell carcinoma (OSCC), which is primarily caused by tobacco and alcohol use, accounts for approximately 400,000 new cases per annum, with the highest incidence rates being found in South Asia and Southeast Asia (India, Pakistan, Sri Lanka and Taiwan) (Warnakulasuriya, 2009). Nasopharyngeal carcinoma (NPC) is rare in most parts of the world but it is particularly prevalent in southern China and South East Asia where it is strongly associated with Epstein-Barr virus infection (Tsang & Tsao, 2015). The prognosis for patients diagnosed with HNSCC is poor, for a variety of reasons that include late presentation. The identification of biomarkers that can be used for screening, diagnosis and prognostication, therefore, would be beneficial clinically.

The measurement of the concentrations of lipid species in biological samples using liquid chromatography-mass spectrometry (LCMS), lipidomics, can be used to identify differences in lipid profiles in the plasma of cancer patients. For example, Lee and colleagues recently used an untargeted approach to profile plasma lipid levels in five different cancers and showed that high abundance lipid species might have value as cancer-specific lipid biomarkers (Lee, Lee & Moon, 2019). Such studies in HNSCC are very limited, but one study has shown that sphingolipid levels were increased in the plasma of OSCC patients compared to non-cancerous controls, whilst the levels of a number of other lipid species, including glycerophospholipids, such as glycerolphosphocholines, glycerophosphoethanolamines, glycerophosphoglycerols and lycophosphocholines levels were decreased (Wang et al., 2017).

Lysophosphatidic acid (LPA) is a lipid mediator formed principally by the action of autotaxin (ATX) (Aoki, 2004), that binds to a subfamily of G protein-coupled cell surface receptors (LPAR1-6) to regulate growth, survival, invasion and angiogenesis (Yung, Stoddard & Chun, 2014). Deregulation of the ATX-LPA-LPAR axis has been reported in many different tumour types and is known to promote tumour progression, metastasis and tumour cell survival (Tigyi et al., 2019). The levels of plasma LPA has been proposed as a potential biomarker in ovarian cancer and other gynaecological cancers (Cao et al., 2015; Zhang et al., 2015). Interestingly, there is some evidence to indicate that levels of different LPA species may also vary during tumorigenesis because 18:2 LPA levels were found to be decreased in the plasma of patients with gastric cancer (Lee, Lee & Moon, 2019), whilst in hepatocellular carcinoma, plasma 18:2 levels were similarly lower, but 20:4 LPA levels were elevated (Skill et al., 2013). Reports describing the role of LPA in HNSCC are limited, however, in OSCC it has been shown that LPA enhances cell proliferation and motility and these effects are regulated by LPAR3 and LPAR4 (Brusevold et al., 2014; Matayoshi et al., 2013). In NPC, we have shown that LPA enhances the migration of NPC cells and also inhibits the activity of EBV-specific cytotoxic T cells (Yap et al., 2015). However, studies measuring levels of LPA in the plasma of HNSCC patients have not been reported.

The major LPA species found in human plasma have been shown to be LPA 16:0, 17:0, 18:0, 18:1, 18:2 and 20:4 (Baker et al., 2000) and these species have been included in previous studies that examined LPA levels in plasma from patients with breast and ovarian cancers (Murph et al., 2007; Shan et al., 2008). Therefore, in the present study, we analysed the concentrations of these LPA species in plasma samples from patients with OSCC and NPC, together with those from control subjects without a history of cancer, using a modified and simple extraction method for small volume samples, followed by LCMS/MS.

## Materials & Methods

### Chemicals and reagents

16:0 LPA, 17:0 LPA, 18:0 LPA, 18:1 LPA, 18:2 LPA and 20:4 LPA were purchased from Avanti Polar Lipids (Alabaster, AL, USA). HPLC-grade solvents were purchased from Fisher Scientific (Pittsburgh, PA, USA). Plasma for standards was obtained from the University of Malaya Medical Centre (UMMC) blood bank. Activated charcoal was purchased from R & M Chemicals (Essex, UK).

### Plasma samples

Plasma samples of OSCC patients and control subjects (a total of 120 samples) were obtained from the Malaysian Oral Cancer Database and Tissue Bank System (MOCCTBS) and managed by the Oral Cancer Research and Coordinating Centre (OCRCC), University of Malaya. Plasma from control subjects were collected concurrently from healthy individuals with no history of cancer. A second set of plasma samples was collected from NPC patients and control subjects (a total of 98 samples) from a public hospital. All cancers were diagnosed by histopathological analysis of biopsy specimens. Information regarding the gender, age, risk factors and cancer stage are shown in Table 1. All participants provided written informed consent. Ethical approval for this study was obtained from the Medical Ethics Committee, Faculty of Malaya (DF OB1403/0009L) and the Medical Research Ethic Committee of the Ministry of Health Malaysia (KKM/NIHSEC/08/0804/P-12-182).

**Table 1:** Details and characteristics of OSCC, NPC and control subjects.

**Characteristics**	****	**Control (*n* = 40) (%)**	**OSCC (*n* = 80) (%)**	**Control (*n* = 49) (%)**	**NPC(*n* = 49) (%)**
Gender	Male	19 (47.50)	29 (36.25)	42 (85.71)	42 (85.71)
	Female	21 (52.50)	51 (63.75)	7 (14.29)	7 (14.29)
Age (years)	≤50	27 (67.50)	22 (27.50)	23 (46.94)	28 (57.14)
	≥50	13 (32.50)	58 (72.5)	26 (53.06)	21 (42.86)
Smoking status	Smokers	3(7.50)	27 (33.75)	N/A	N/A
	Non-smokers	37 (92.50)	53 (66.25)	N/A	N/A
Betel chewing status	Chewers	3 (7.50)	37 (46.25)	N/A	N/A
	Non- chewers	37 (92.50)	43 (53.75)	N/A	N/A
Drinking	Drinkers	8 (20.00)	20 (25.00)	N/A	N/A
	Non-drinkers	32 (80.00)	60 (75.00)	N/A	N/A
Stage	Early (I&II)	N/A	29 (36.25)	N/A	N/A
	Advanced (III&IV)	N/A	51 (63.75)	N/A	N/A

For sample collection, whole blood (three mL) was obtained preoperatively in EDTA tubes and transported on ice at 4 °C prior to processing. Samples were centrifuged at 1500 rpm for 15 min. Plasma was immediately aliquoted into micro-tubes (0.3 mL each tube) and stored at −80 °C until preparation for mass spectrometry analysis.

### Sample preparation for LC-MS/MS

All LPA stocks and internal standard (IS; 17:0 LPA) were prepared in 50% (v/v) ethanol at a concentration of 1.0 mg/mL, which is equivalent to 1,000 parts per million (ppm). For the preparation of calibration curves for quantitative analysis, LPA stocks were diluted in blank plasma (without any endogenous LPA). Blank plasma was prepared by adding 10% (w/v) activated charcoal to plasma to deplete LPA. LPA depletion was monitored daily using LCMS. The activated charcoal was replaced with fresh activated charcoal at day 4. Complete depletion was achieved after 9 days. Blank plasma samples were also treated with three freeze/thaw cycles. The entire procedure was performed with continuous shaking on ice. The plasma-charcoal mixture was centrifuged at 16000 × g for 10 min and the supernatant was aspirated and filtered using a 0.22 µm nylon filter. The filtrate (blank plasma) was then used to prepare the LPA standards for the calibration curves.

A diluted working stock of 2000 ppb was finally prepared in blank plasma to prepare 100 ppb, 200 ppb, 350 ppb, 500 ppc, 700 ppb, 1000 ppb and 1500 ppb as the standard curve for quantification. Quality control (QC) samples were prepared at 300 ppb (QC1), 750 ppb (QC2) and 1350 ppb (QC3). Calibration standards and QC samples were prepared fresh daily. 17:0 LPA was added to all samples as an internal standard (IS) at a final concentration of 5ppm. Representative MRM chromatograms showing retention times of the individual LPAs added to blank plasma are shown in Fig. S1.

### LPA extraction from plasma

LPA was extracted from the plasma of OSCC and NPC patients and controls, as described previously (Zhao & Xu, 2010) with minor modifications. LPA standards and QC samples were extracted using the same extraction method. Briefly, 10 µL of plasma were added to 300 µL of methanol (MeOH) together with 20 µL of 500 ppb of 17:0 LPA (non-naturally occurring LPA species) as the internal standard. Samples were vortexed and incubated on ice for 10 min and later centrifuged at 16000 × g for 10 min at room temperature. 180 µL of the supernatant were transferred to a fresh glass vial and subjected to LC-MS/MS analysis.

### Liquid chromatography with tandem mass spectrometry (LCMS/MS)

The LPA species were separated by reversed-phase liquid chromatography using a C6 Phenyl analytical column (Gemini, particle size 5 µm, pore size 110 Å, 150 × 2.0 mm, Phenomenex, Torrance, CA, USA) and an injection volume of 10 µL with a 10 min separation time. The mobile phase consisted of H20 containing 0.01% NH3 and Acetonitrile: Methanol (50:50, v/v) and was delivered at a flow rate of 0.5 mL/min. The LC-MS/MS system comprised of an Applied Bioscience (AB) SCIEX QTRAP 5500 mass spectrometer, equipped with an ultra-fast liquid chromatography Shimadzu LC system and auto sampler (Shimadzu UFLC XR). The electrospray ion source was run in a negative ionization mode. Quantification was performed in the multiple reaction-monitoring (MRM) mode. Calibration standards and QC samples were extracted and quantitated daily together with approximately 20–30 plasma samples. Quantitative data measured against a linear standard curve was analysed with Analyst data acquisition system.

### Method validation

The analysis method was validated according to the bioanalytical method validation procedures outlined by the USFDA (FDA, 2018). A set of five replicates of each QC (QC1, QC2 and QC3) were prepared and analysed each day to determine the intra-day or inter-day precision and accuracy of the method. These analyses were repeated daily over three days using freshly prepared calibration curves. Freeze and thaw stability was tested with QC1 and QC3 where all samples were kept frozen at −40 °C for 25 mins and thawed at room temperature for three cycles. Analysis was carried out for every cycle. Sample stability was tested after 4 and 6 h at room temperature and at 4 °C. Post-preparative auto sampler stability was determined by reinjection of QC1 and QC3 after 4 and 6 h following the initial injection. Long term stability of QC samples was analysed after 48 days. Intra-day accuracy and precision were calculated from % bias (% (measured –theoretical)/theoretical concentration] and relative standard deviation [%RSD = % standard deviation/mean] respectively for the QC points. Inter-day accuracy was calculated similarly for each QC points from 3 validation runs. Absolute recovery was calculated by comparing the peak area of QC samples in plasma to the peak area of samples in untreated neat solution (methanol).

### Statistical analyses

Bivariate analysis was carried out in order to understand any association between two variables (LPA species). Box plots describe the average values of median, minimum, maximum and the variability of the datasets using the first and third quartiles. The Kruskall–Wallis test was used in testing the differences in LPA plasma concentrations between controls, OSCC (early and advanced stage) and NPC. Correlation between pairwise LPA species was shown using scatterplots. Visual presentation using heat maps was drawn to show how all LPA values varied across all samples. Bivariate analyses were performed using the R programming language. Multivariate analysis (Principal Component Analysis (PCA) was performed using the SIMCA-P v12 (UMETRICS) software. PCA was performed on all five LPA species for all 218 samples from the two groups.

## Results

### Levels and profile of LPA species in plasma from OSCC and NPC patients

**Figure 1:**
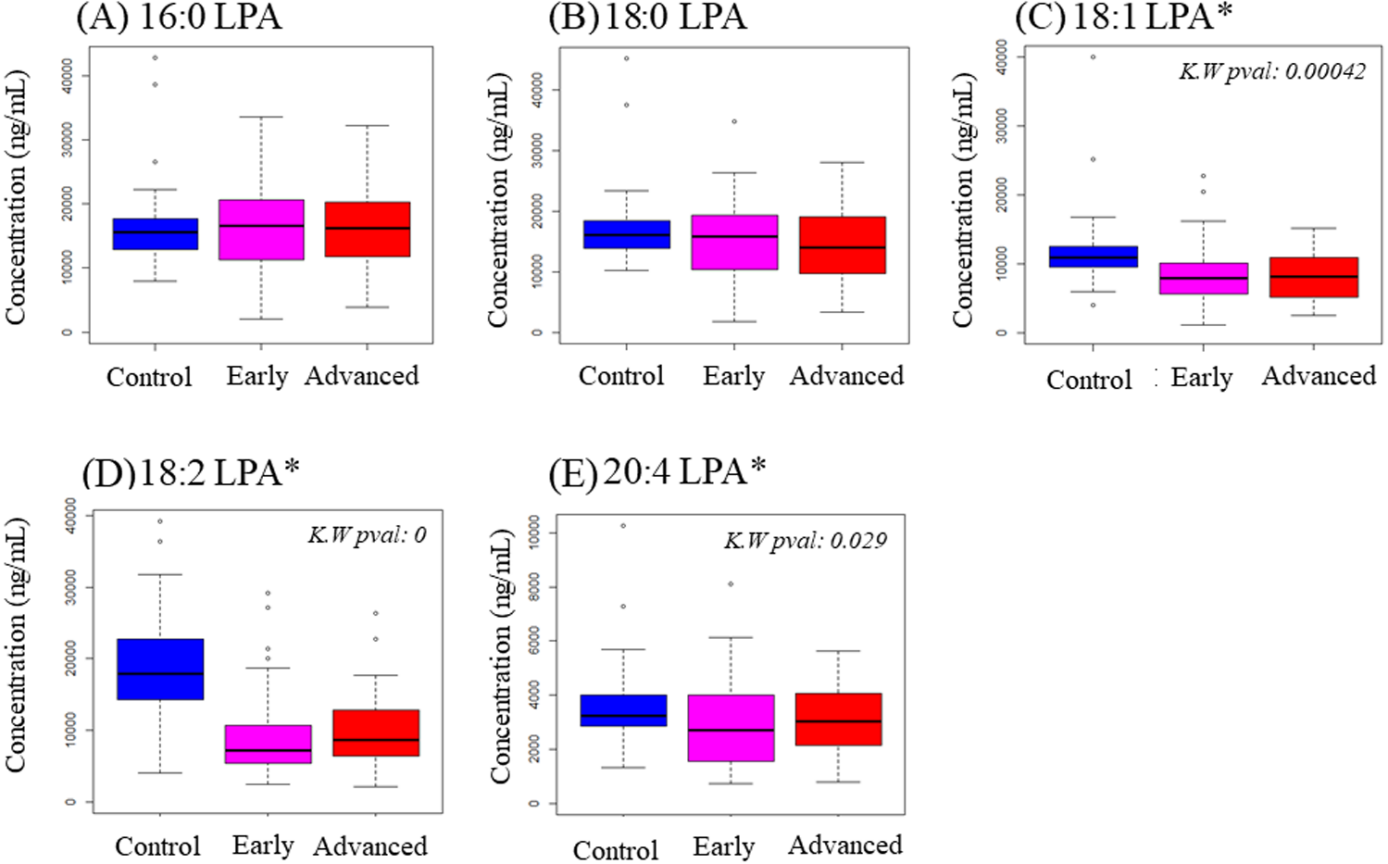
Concentration of five LPA species in plasma samples from OSCC patients and controls. Data are presented using box plots for (A) 16.0 LPA, (B) 18.0 LPA, (C) 18.1 LPA, (D) 18.2 LPA and (E) 20.4 LPA. There were significant differences between the three groups for three LPA species (18:1 LPA, 18:2 LPA and 20:4 LPA). K.W. represents Kruskal–Wallis test and * indicates *p* < 0.05. *y*-axis represents concentrations of LPA in plasma in ng/mL.

**Figure 2:**
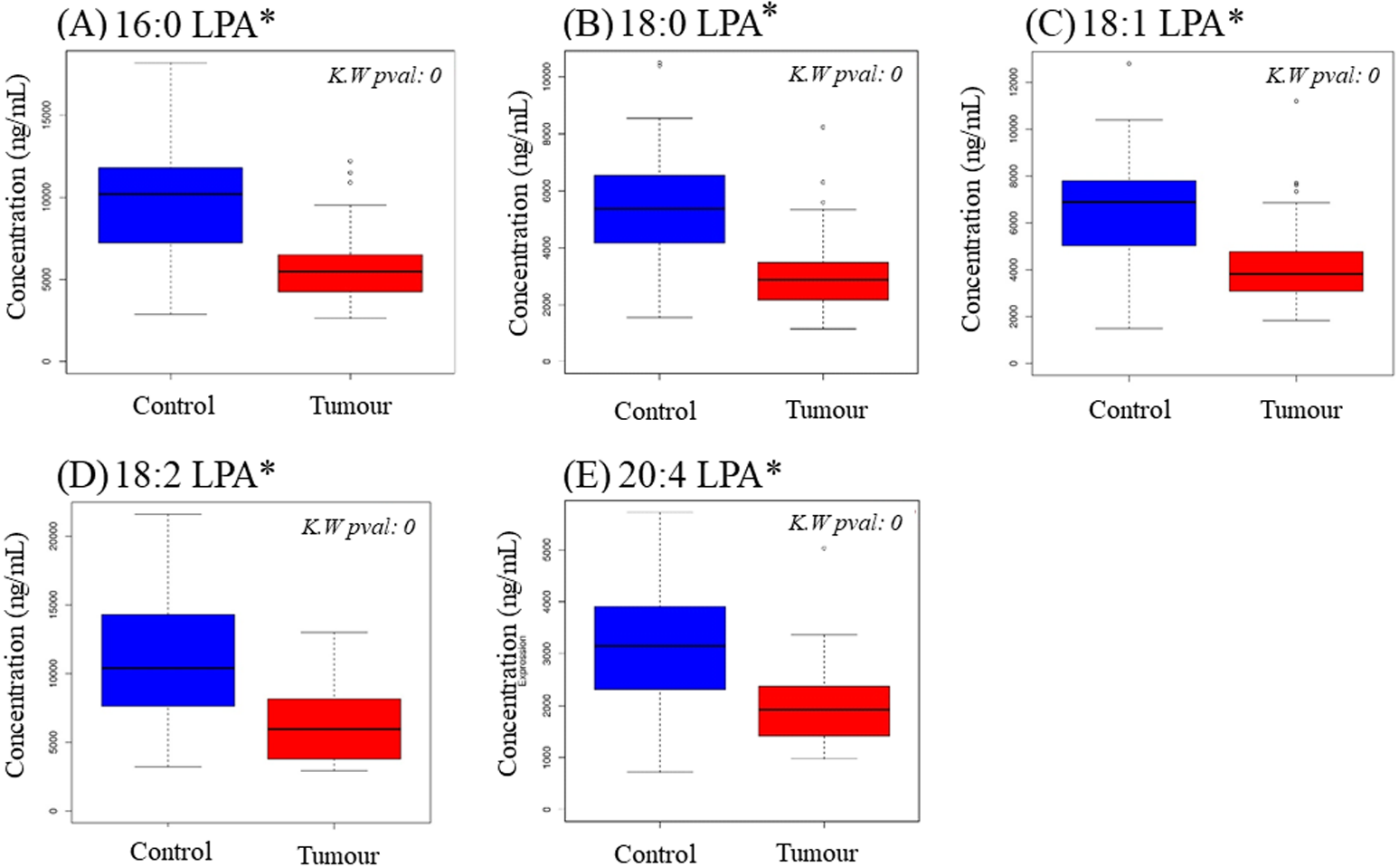
Concentration of five LPA species in plasma samples from NPC patients and controls. Data are presented using box plots for (A) 16.0 LPA, (B), 18.0 LPA, (C) 18.1 LPA, (D) 18.2 LPA and (E) 20.4 LPA. There were significant differences between normal and NPC samples for all LPA species. K.W. represents Kruskal–Wallis test and * indicates *p* < 0.05.

To examine possible variations in individual LPA levels, LPA species were quantified in plasma samples from OSCC and NPC patients together with control subjects using LCMS/MS. Total LPA levels in control plasma were higher than those in plasma from cancer patients for both OSCC (Table 2) and NPC (Table 3). The levels of individual LPA species (16:0, 18:0, 18:1, 18:2 and 20:4) in plasma from cancer patients were compared to those in healthy control individuals using boxplots and are shown in Fig. 1 (OSCC) and Fig. 2 (NPC). For OSCC, the levels of three individual LPAs (18:1 LPA, 18:2 LPA and 20:4 LPA) were significantly different between the three groups (normal, early and advanced) (*p* < 0.05; Fig. 1). Post hoc tests revealed that both 18:1 LPA and 18:2 LPA levels were significantly lower in the early and advanced cancer groups compared with normal controls and 20:4 LPA levels were significantly lower in the early OSCC group than in the controls (Table 4). The levels of all LPA species examined were lower in plasma from NPC patients compared with the controls (*p* < 0.05). (Fig. 2; Table 3). In the present study, the relative abundance of the LPA species in the normal plasma samples was similar to previous reports, with the order of abundance being LPA 18:2>16:0>18:0>18:1>20:4 (Baker et al., 2002; Shan et al., 2008). Interestingly, in both cancer groups the relative order of abundance for both cancer types was different from the controls with LPA 16:0>18:0>18:2>18:1>20:4.

**Figure 3:**
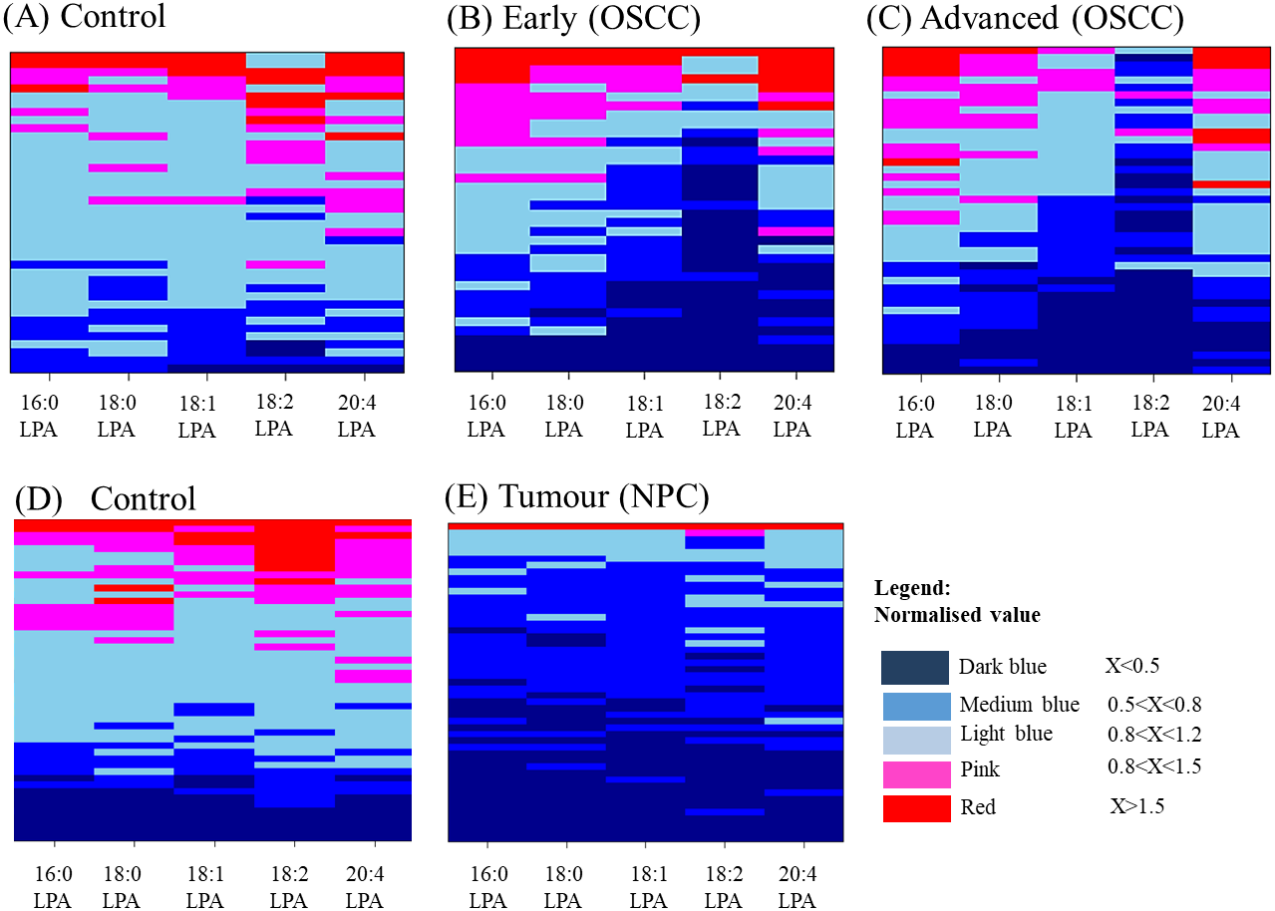
Heatmaps showing the distribution of LPA levels in plasma from OSCC and NPC patients with their respective and controls. Each LPA species was shown in a separate column and samples shown in rows for OSCCs and controls (A–C) and NPCs (D and E). Samples were ordered from top to bottom by decreasing value of total LPA amounts. Each sample was represented by five LPA values (16:0 LPA, 18:0 LPA, 18:1 LPA, 18:2 LPA and 20:4 LPA) presented in aligned horizontal boxes. Each LPA data was normalised with normal median value of each individual LPA. The colours indicate the ratio between the two values starting from the lowest range (dark blue) to the highest range (red). Light blue box represents the median value for normal sample.

The distributions of LPA in plasma samples using normalized values of all five LPA concentrations were highlighted and visualised by the construction of Heatmaps (Fig. 3). For control oral samples, the majority of samples demonstrated LPA values similar or close to the normal median value (0.8<X<1.2). For both early and advanced OSCC groups, a different profile was observed with a subset of samples for which LPA concentrations were generally lower than in normal samples. Similar results were obtained for NPC, with all LPA concentrations being generally lower in most of the tumours.

### Correlation between individual LPA species

**Figure 4:**
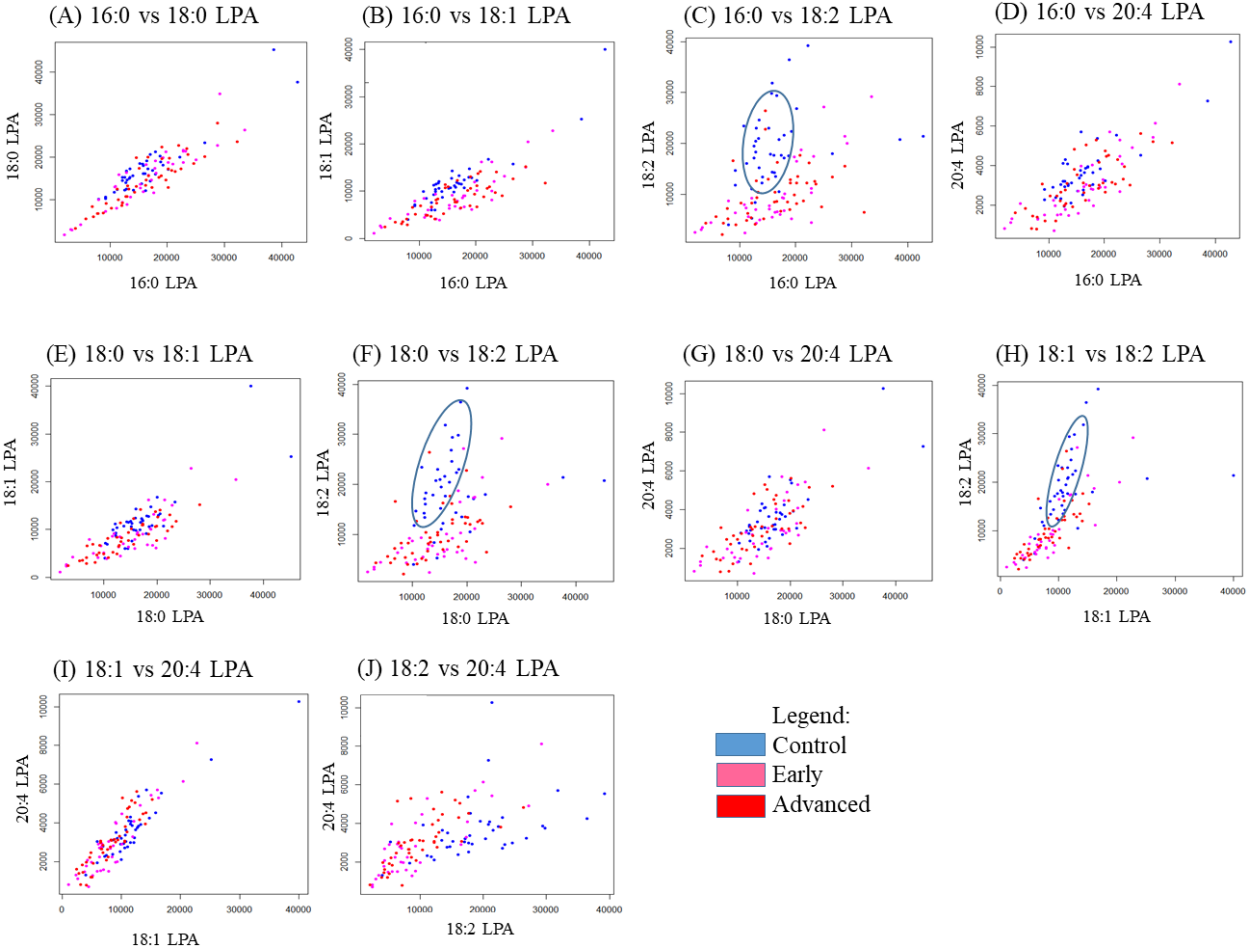
Scatterplots of LPA species that have distinctive differences between the three OSCC groups (control, early and advanced). Scatter plots were generated for all possible pairs of LPA species examined in plasma from patients with early and late OSCC plus controls (A–J). For three pairs of LPA species, 16:0 LPA versus 18:2 LPA (C), 18:0 LPA versus 18:2 LPA (F) and 18:1 LPA versus 18:2 LPA (H) the data points from control samples clustered together as indicated by the blue ellipse.

**Figure 5:**
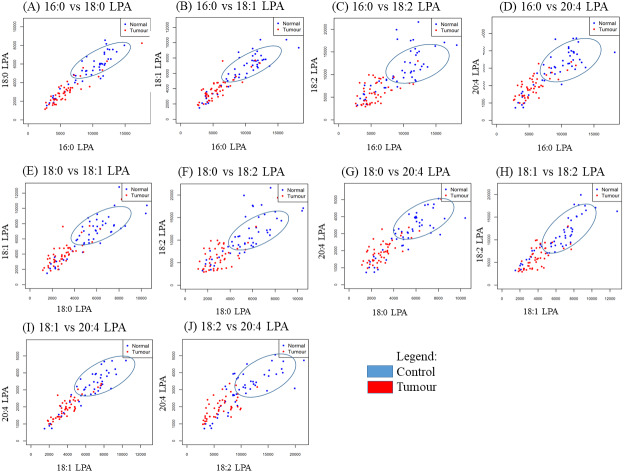
Scatterplots of paired LPA species in plasma from patients with NPC and controls. Scatter plots were generated for all possible pairs of LPA species examined in plasma from NPC patients and controls (A–J). For all pairs, the data points from the controls clustered together, as indicated by the blue ellipse.

In order to study the relationship between each pair of LPA species, correlation studies were conducted using Pearson correlation tests (Table 5). For OSCC, strong to moderate positive correlations were observed between the three groups (control, early and advanced) for all LPA species (Table 5). A similar result was observed in the NPC samples with strong positive correlations observed between the two sample groups (control, tumour) for all LPA species (Table 6). To further investigate these correlations, scatterplots were generated for all possible pairings of the five LPA species measured. The results for the OSCC samples are shown in Fig. 4. For three pairs of LPA species (18:2 LPA paired with 16:0 LPA, 18:0 LPA or 18:1 LPA), the data points for normal samples were clustered together allowing possible differentiation between control and cancer groups (both early and advanced). For NPC samples, clustering of control samples was observed in all plots (Fig. 5).

**Table 2:** LPA levels in plasma of control subjects and OSCC patients.

	**Plasma samples**	**N (number of samples)**	**LPA concentration** Mean(µM) ± SEM
TOTAL	Control	40.00	146.77 ± 7.98
LPA	Early (stage I and II)	40.00	112.98 ± 8.79
	Advanced (stage III and IV)	40.00	113.96 ± 6.54
16:0 LPA	Control	40.00	37.92 ± 2.45
	Early (stage I and II)	40.00	36.66 ± 2.69
	Advanced (stage III and IV)	40.00	37.24 ± 2.33
18:0 LPA	Control	40.00	37.20 ± 2.23
	Early (stage I and II)	40.00	31.85 ± 2.39
	Advanced (stage III and IV)	40.00	31.46 ± 1.95
18:1 LPA	Control	40.00	24.59 ± 1.89
	Early (stage I and II)	40.00	18.05 ± 1.59
	Advanced (stage III and IV)	40.00	17.61 ± 1.14
18:2 LPA	Control	40.00	39.79 ± 2.48
	Early (stage I and II)	40.00	20.96 ± 2.24
	Advanced (stage III and IV)	40.00	21.51 ± 1.68
20:4 LPA	Control	40.00	7.26 ± 0.49
	Early (stage I and II)	40.00	5.74 ± 0.53
	Advanced (stage III and IV)	40.00	6.13 ± 0.41

**Table 3:** LPA levels in plasma of control subjects and NPC patients.

	**Plasma samples**	**N (number of samples)**	**LPA concentration** Mean (µM) ± SEM		
Total LPA	Control	49	79.59 ± 3.97
	NPC	49	45.93 ± 2.72
LPA 16.0	Control	49	21.93 ± 1.18
	NPC	49	14.03 ± 0.90
LPA 18.0	Control	49	11.71 ± 0.66
	NPC	49	6.81 ± 0.41
LPA 18.1	Control	49	13.22 ± 0.74
	NPC	49	8.65 ± 0.51
LPA 18.2	Control	49	22.62 ± 1.33
	NPC	49	13.46 ± 0.87
LPA 20.4	Control	49	6.09 ± 0.34
	NPC	49	4.03 ± 0.21

### Clustering of samples by all LPA values

**Figure 6:**
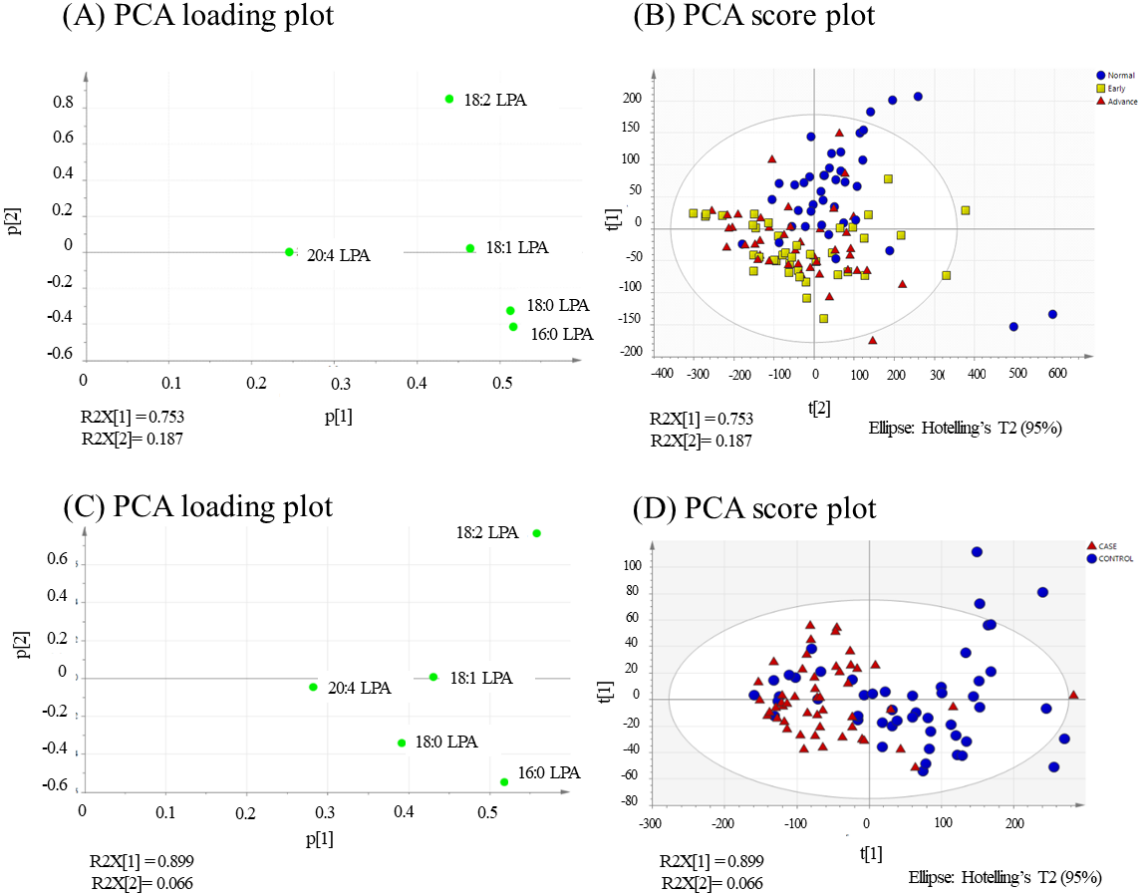
PCA loading and score plots for OSCC and NPC. Values for the loading plots were from all the five LPA species analysed are shown for OSCC (A and B) and NPV C and D). Overview of PCA score plot obtained from all normal (blue), early (yellow) and advanced (red) samples.

PCA was performed to further evaluate the separation between normal and OSCC samples according to their LPA values. The concentrations of five LPA species (16:0 LPA, 18:0 LPA, 18:1 LPA, 18:2 LPA and 20:4 LPA) were considered in this analysis. In PCA analysis, total percentage of PCA variation between samples of the first two principal components was 94.0% with values of 75.3% and 18.7% for the respective components (Fig. 6A). The PCA score plot of these first two components provided a good separation of the normal samples from both early and advanced tumour samples. The loading plot graph shows the coefficients of each variable for the first component versus the second component. This identifies which variables have the largest effect on each component. A scrutiny of the PCA loading plot, showed that all LPA have positive loadings on component 1 (*x*-axis) with the strongest influence from 16:0 LPA and 18:0 LPA. As for component 2 (*y*-axis), 18:2 LPA has the highest influence among the variables.

In NPC samples (Fig. 6B), the total percentage of PCA variation between samples of the first two principal components was 96.5% with values of 89.9% and 6.6% for the respective components. The PCA score plot showed that these two components provided a good separation between control and tumour samples. Similar to OSCC, the NPC showed that all LPA have positive loadings on component 1 (*x*-axis) with the strongest influence from 16:0 LPA and 18:2 LPA. As for component 2 (*y*-axis), 18:2 LPA has the highest influence among the variables.

**Table 4:** Kruskal–Wallis post-hoc test comparison of 18:1 LPA, 18:2 LPA and 20:4 LPA levels.

**Pairwise comparison between groups**	**18:1 LPA**	**18:2 LPA**	**20:4 LPA**
Early-Advanced	1.0	1.0	0.927
Early-Control	0.002*	<0.05*	0.027*
Advanced-Control	0.002*	<0.05*	0.267

## Discussion

HNSCC is a significant world health problem that is particularly prevalent in southern China and South East Asia. OSCC and NPC are distinct types of HNSCC with distinct aetiologies. The prognosis for patients with OSCC and NPC is poor and this is in part due to late presentation and disease recurrence. The identification of clinically useful biomarkers would be a significant advance to aid in patient management. In the present study, we measured the levels of five LPA species (16:0 LPA, 18:0 LPA, 18:1 LPA, 18:2 LPA and 20:4 LPA) in the plasma of patients with HNSCC (OSCC and NPC) and control subjects using LCMS/MS. These species were selected because they have previously been shown to be the most abundant species in human blood (Baker et al., 2000).

In the present study, LPA was extracted using a modified and simple method (Zhao & Xu, 2010) by precipitating blood proteins in methanol and has no solvent evaporation step. This allows the extracted lipids to be analysed directly and is therefore suitable for small sample volumes. The results of our study show that the levels of three LPA species (18:1 LPA, 18:2 LPA and 20:4 LPA) were significantly lower in the plasma of OSCC patients (both early and late stage), whilst the concentrations of all five LPA species examined were significantly lower in plasma from NPC patients. Our results differ from some previous studies in other cancer types that showed that LPA levels were markedly elevated in biological fluids, such as the ascites fluid of ovarian cancer patients and the blood of other gynaecological cancers (Xu et al., 1998; Yoon, Kim & Cho, 2003) and multiple myeloma (Sasagawa et al., 1999). There have been some contradictory reports, however, as Baker and colleagues reported that there were no significant differences in the concentrations of either individual LPA values or total LPA in the plasma of ovarian cancer patients compared to normal controls (Baker et al., 2002) and, recently, it was reported that LPA levels were lower in the plasma of ovarian cancer patients (Yagi et al., 2019). These discrepancies have been suggested to be due to the fact that LPA levels in plasma can be artificially increased during sample handling and processing (Yagi et al., 2019). Our data would indicate that total plasma LPA levels *per se* are not biomarkers for HNSCC. The reasons why LPA levels are lower in the plasma of OSCC and NPC patients are unclear. We have shown that the expression of PPAP2 that encodes for lipid phosphate phosphatase-1 (LPP1) is down-regulated in OSCCs and ENPP2 (the gene that encodes for ATX) is elevated in a subset of OSCC tissues (Abdul Rahman et al., manuscript in revision). Together, this would likely lead to a localized increase in LPA within the tumours, which would not be surprising considering the tumour promoting properties of LPA. However, plasma levels of LPA are more likely to be regulated by ATX expressed by non-malignant cells during reprogramming of the tumour microenvironment or by circulating immune cells (Tigyi et al., 2019). Further, LPA is rapidly degraded by membrane-bound phosphatases (Moolenaar & Perrakis, 2011) to keep plasma LPA concentrations low and this may be altered during tumorigenesis. Our data indicate that plasma LPA levels are reduced in two distinct types of HNSCC, namely OSCC and NPC. Even though these datasets represent different cohorts of patients, it is cautionary to note that further studies are needed to verify our data and investigate the relevant biological mechanisms.

**Table 5:** Correlation between two LPA species in OSCC samples.

**LPA species**	**16:0 LPA**	**18:0 LPA**	**18:1 LPA**	**18:2 LPA**	**20:4 LPA**
16:0 LPA		0.901^**^	0.819^**^	0.409^**^	0.816^**^
18:0 LPA	0.901^**^		0.833^**^	0.467^**^	0.768^**^
18:1 LPA	0.819^**^	0.833^**^		0.666^**^	0.908^**^
18:2 LPA	0.409^**^	0.467^**^	0.666^**^		0.628^**^
20:4 LPA	0.816^**^	0.768^**^	0.908^**^	0.628^**^	

**Notes.**

Pearson correlation test was performed using values for two LPA species in each sample. The table shows Pearson correlation coefficients (*r*) for the LPA species. Dark grey box, strong correlation (*r*: 0.7–1.0); light grey box, moderate correlation (*r*: 0.3–0.7); white box. No correlation. Only positive correlations were observed in all analysis.

** *p* < 0.01.

**Table 6:** Correlation between two LPA species in NPC samples.

**LPA species**	**16:0 LPA**	**18:0 LPA**	**18:1 LPA**	**18:2 LPA**	**20:4 LPA**
**16:0 LPA**		0.943^**^	0.918^**^	0.803^**^	0.880^**^
**18:0 LPA**	0.943^**^		0.886^**^	0.813^**^	0.873^**^
**18:1 LPA**	0.918^**^	0.886^**^		0.897^**^	0.943^**^
**18:2 LPA**	0.803^**^	0.813^**^	0.897^**^		0.844^**^
**20:4 LPA**	0.880^**^	0.873^**^	0.943^**^	0.844^**^	

**Notes.**

Pearson correlation test was performed using values for two LPA species in each sample. The table shows Pearson correlation coefficients (*r*) for the LPA species. Dark grey box, strong correlation (*r*: 0.7–1.0); light grey box, moderate correlation (*r*: 0.3-0.7); white box. No correlation. Only positive correlations were observed in all analysis.

** *p* < 0.01.

In the present study, the relative of abundance of LPA species in the normal plasma samples was similar to previous reports, with the order of abundance being LPA 18:2>16:0>18:0>18:1>20:4 (Baker et al., 2002; Shan et al., 2008). Interestingly, the profiles for both cancer groups were different from the controls with the order of abundance for both cancer types being LPA 16:0>18:0>18:2>18:1>20:4. These data indicate that the individual LPA species might play different roles during tumorigenesis. In this regard, correlation studies were conducted using Pearson correlation tests and the results showed that normal and tumour samples could be separated based on the levels of certain pairs of LPA species. These data were confirmed using PCA which showed a good separation of normal and cancer groups with 16.0 and 18.0 LPA having the strongest influence. Studies on the biological effects of the individual LPA species in cancer are limited, but differences have been reported. For example, unsaturated LPAs (18:1 LPA and 20:4 LPA) were more potent than saturated LPA (16:0 LPA) in stimulating LPAR3-mediated cell growth in an ovarian cancer cell line (SKOV-3) (Hurst & Hooks, 2009). Further, unsaturated LPA species were reported to be more efficient chemo-attractants than unsaturated LPAs for immature mouse dendritic cells, an effect also mediated by LPAR3 (Chan etal , 2007). Further studies to investigate the roles of specific LPA species in HNSCC and other cancers are warranted.

## Conclusions

We have profiled the concentration of the five major LPA species in the plasma of HNSCC patients (OSCC and NPC) and compared these to the profiles from normal control individuals. We show for the first time that LPA levels are lower in HNSCC cancer patients and that the order of abundance of LPA species in plasma was different between cancer patients and control subjects. These results indicate the potential of selected LPA species as potential biomarkers for OSCC and NPC and highlight the need to investigate the roles of specific LPA species in cancer and other diseases.

##  Supplemental Information

10.7717/peerj.9304/supp-1 Figure S1 Representative MRM chromatograms of individual LPAs Individual retention times for each LPA are as follows: 16:0 LPA-0.642min, 17:0 LPA-0.636min, 18:0 LPA-0.661min, 18:1 LPA-0.644min, 18:2 LPA-0.631 and 20:4 LPA-0.630min. 17:0 LPA was the internal standard incorporated in all standard mixtures and samples for normalization of all runs. The overall run time for chromatography was 5 mins.

10.7717/peerj.9304/supp-2 Data S1 Raw data for the LPA values for NPC patients and controls

10.7717/peerj.9304/supp-3 Data S2 Raw data for LPA levels in OSCC patients and controls

## Additional Information and Declarations

### Data Availability

The following information was supplied regarding data availability: The raw measurements of LPA levels are available in the Supplementary Files.
