# Supplementary material for: Profiling lysophosphatidic acid levels in plasma from head and neck cancer patients
Source: PeerJ. 2020 Jun 5;8:e9304. doi: 10.7717/peerj.9304 (PMC7278886; doi:10.7717/peerj.9304)
Supplement: Figure S1 — Individual retention times for each LPA are as follows: 16:0 LPA-0.642min, 17:0 LPA-0.636min, 18:0 LPA-0.661min, 18:1 LPA-0.644min, 18:2 LPA-0.631 and 20:4 LPA-0.630min. 17:0 LPA was the internal standard incorporated in all standard mixtures and samples for normalization of all runs. The overall run time for chromatography was 5 mins. [file peerj-08-9304-s001.pdf]

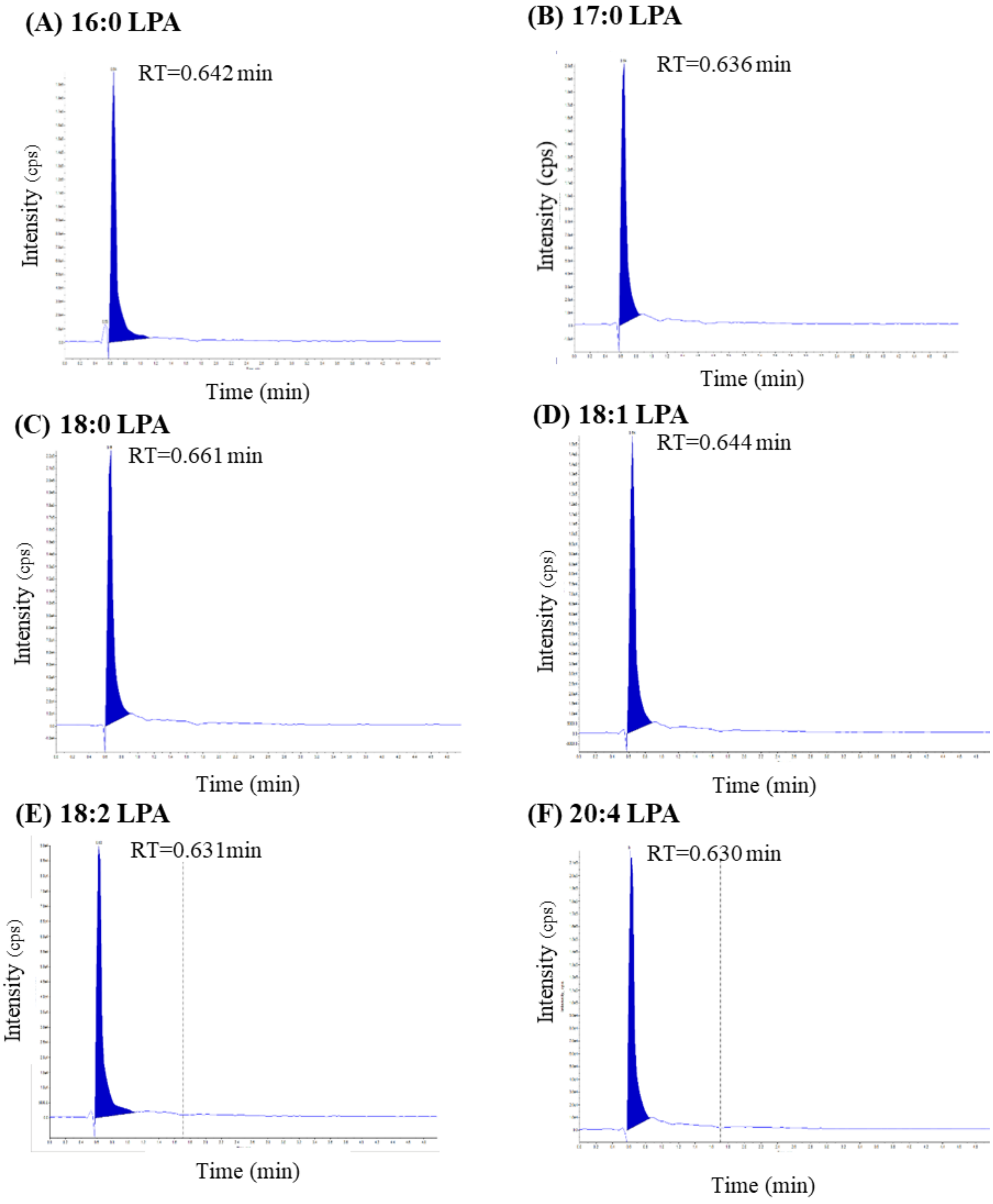

Individual retention times for each LPA are as follows: 16:0 LPA-0.642min, 17:0 LPA-0.636min, 18:0 LPA-0.661min, 18:1 LPA-0.644min, 18:2 LPA-0.631 and 20:4 LPA-0.630min. 17:0 LPA was the internal standard incorporated in all standard mixtures and samples for normalization of all runs. The overall run time for chromatography was 5 mins.
